# Unveiling a Selective Mechanism for the Inhibition of α-Synuclein Aggregation by β-Synuclein

**DOI:** 10.3390/ijms19020334

**Published:** 2018-01-24

**Authors:** Andre Leitao, Akshay Bhumkar, Dominic J. B. Hunter, Yann Gambin, Emma Sierecki

**Affiliations:** 1 European Molecular Biology Laboratory (EMBL), Australia Node in Single Molecule Science, Sydney NSW 2031, Australia; a.leitao@unsw.edu.au (A.L.); a.bhumkar@unsw.edu.au (A.B.); d.hunter@imb.uq.edu.au (D.J.B.H.); 2 School of Medical Sciences, The University of New South Wales, Sydney NSW 2031, Australia; 3 Institute for Molecular Bioscience, The University of Queensland, St Lucia QLD 4076, Australia

**Keywords:** α-synuclein, β-synuclein, Parkinson’s disease, protein oligomerization, single molecule spectroscopy, number and brightness analysis, two-color coincidence

## Abstract

α-Synuclein (αS) is an intrinsically disordered protein that is associated with Parkinson’s disease (PD) through its ability to self-assemble into oligomers and fibrils. Inhibition of this oligomerization cascade is an interesting approach to developing therapeutical strategies and β-synuclein (βS) has been described as a natural negative regulator of this process. However, the biological background and molecular mechanisms by which this inhibition occurs is unclear. Herein, we focused on assessing the effect of βS on the aggregation of five αS pathological mutants linked to early-onset PD (A30P, E46K, H50Q, G51D and A53T). By coupling single molecule fluorescence spectroscopy to a cell-free protein expression system, we validated the ability of βS to act as a chaperone of αS, effectively inhibiting its aggregation. Interestingly, we found that βS does so in a selective manner, i.e., is a more effective inhibitor for certain αS pathological mutants—A30P and G51D—as compared to E46K, H50Q and A53T. Moreover, two-color coincidence experiments proved that this discrepancy is due to a preferential incorporation of βS into smaller oligomers of αS. This was validated by showing that the chaperoning effect was lost when proteins were mixed after being expressed individually. This study highlights the potential of fluorescence spectroscopy to deconstruct αS aggregation cascade and its interplay with βS.

## 1. Introduction

Pathological protein aggregation is a poorly understood phenomenon that lies at the root of several neurodegenerative diseases including Parkinson’s, Alzheimer’s, Huntington’s and Creutzfeld–Jakob diseases. For each of these diseases, one or more proteins have been shown to self-assemble into highly-ordered fibrils with further accumulation into amyloid deposits. In Parkinson’s disease (PD), these intracellular deposits are called Lewy Bodies and are primarily composed of the presynaptic protein α-Synuclein (αS) [1].

α-Synuclein (αS) is amongst some of the most studied aggregation-prone proteins in neurodegeneration, mostly due to its involvement in the pathogenesis of Parkinson’s disease (PD) and other disorders such as Dementia with Lewy Bodies (DLB) or Multiple Systems Atrophy (MSA), all of which are thus generically designated as synucleinopathies [2]. αS is a small 140 amino-acid long protein, encoded by the gene *SNCA*, that predominantly localizes at the presynaptic terminals of neurons, where it comprises 1% of all cytosolic proteins. Although its exact biological function at the presynapse has not been fully elucidated, αS is often associated with or in close proximity of synaptic vesicles, playing an important role in the trafficking of these vesicles, and in the regulation of neurotransmitter exocytosis through membrane remodelling [3,4].

The lack of a defined structured of αS’s monomeric form in solution categorizes it as an intrinsically disordered protein (IDP) and partly explains the challenges associated with understanding its function and aggregation propensity in neurons [5]. In fact, αS’s ability to self-assemble in PD is often depicted as an irreversible cascade of events that leads to the formation of different soluble oligomeric/protofibrillar species and culminates in the formation of insoluble fibrils that ultimately accumulate in Lewy Bodies [1]. Straightforward as this process may seem, it raises a multitude of questions. The succession and roles of each aggregated species are largely unknown and whether different oligomers are formed on- or off-pathway is still controversial. Furthermore, recent studies have shown intermediate oligomeric states to be more toxic than the mature fibrils [6,7], challenging conventional views that have historically associated fibrils as the source of toxicity to neurons. However, whether this mechanism of toxicity is related to gain- or loss-of-function properties of αS aggregates is unclear.

αS’s aminoacid sequence provides us with some clues to its function and aggregation behaviour. The N-terminal part of the polypetide (residues 1–60) is characterized by the presence of six imperfect KTKEGV aminoacid repeats that confer a variation of hydrophobicity with a strict periodicity of 11 residues, a feature that is typical of amphipathic helices in apolipoproteins and critical for lipid or membrane binding [3,5]. The core of the molecule is designated the non-Aβ component (NAC) because it was first reported to be present in amyloid-β deposits in patients with Alzheimer’s disease. It encompasses residues 61–90 and is a highly hydrophobic region that shows high propensity for β-sheet formation, typical of later stages of fibrillation, as opposed to the acidic C-terminal region, which has been shown to be highly soluble [8]. Interconversion between oligomeric forms, which have been shown to be more flexible and characterized by antiparallel β-sheet, and fibrils with a parallel β-sheet core, has been postulated by several authors to be a key step in the progression of aggregation [7,9]. Furthermore, recent data indicate that oligomeric α-synuclein species can spread between cells, and thereby act as seeds, propagating αS pathology [10,11,12,13].

An additional factor that has hindered the understanding of αS’s aggregation is the slow rate and very specific conditions (temperature, pH, lipid content, etc.) at which it occurs. As it happens, five naturally-occurring single-point mutations in the *SNCA* gene have been reported to enhance αS’s oligomerization in vitro and in vivo. In fact, although PD is predominantly sporadic in nature, 5–10% of cases are believed to be familial, leading to earlier-onset forms of the disease. Interestingly, all five different αS missense mutations identified to date in familial forms of PD are contained within the N-terminal domain, and include: A30P, E46K, H50Q, G51D and A53T. Several studies have focused on the aggregation behaviour of these mutant forms of αS and established a much higher propensity to form fibrils as compared to the WT αS [14,15,16,17], attesting the importance of the N-terminal domain in the pathophysiology of PD, particularly in earlier stages of oligomerization. Moreover, striking differences have been identified between the populations of aggregates formed by the different point-mutants regarding β-sheet content, conformational flexibility, intra- and intermolecular interactions and ability to permealize membranes [15,16,18,19,20,21].

Recent studies performed our group [22] using single molecule spectroscopy and a cell free protein expression system have painted a different picture of αS aggregation, where the formation of different aggregate species ultimately defines one of two possible pathways towards fibril formation: E46K, H50Q and A53T rapidly form large fibrils whereas A30P, G51D and WT aggregate less and form smaller objects. Moreover, these groups were shown to be mutually exclusive in their ability to recruit one another’s aggregates.

Finding natural inhibitors of protein aggregation is crucial to both the fundamental understanding of the aggregation process and the development of future therapeutic strategies. Coincidently, αS belongs to a highly conserved family of two other proteins, β- and γ-synuclein, both of which have been reported to inhibit αS fibril formation [23]. However, whilst γ-synuclein is predominantly expressed in sensory- and motor-neurons of the peripheral nervous system and has not been implicated in synucleinopathies, β-synuclein (βS) colocalizes with αS at the presynapse of CNS neurons, where both proteins are expressed at similar levels [24]. βS has been observed to inhibit αS’s aggregation both in vitro [23,25] and in vivo [26,27], raising interest in its potential role as a natural anti-parkinsonian agent. β- and αS show a high degree of sequence homology (61.6%), similar intrinsically disordered nature, as well as ability for lipid binding. Importantly, βS lacks a 11 residue stretch at the NAC domain, which has been hypothesised as being the main cause for its lower propensity to form amyloid fibrils [23]. Up- and down-regulation of αS and βS, respectively, have been correlated with disease onset suggesting that altered relative expression levels of these proteins changes disease progression [28]. Furthermore, α-βS bigenic mice revealed amelioration of neurodegenerative effects compared with single αS transgenic mice [26]. Other in vivo evidence pointing to a neuroprotective role of βS includes the utilization of βS-derived peptides as potential therapeutical strategies revealing phenotype recovery in mice [29] and fly models [30], or the intracerebral injection of lenti-βS virus with subsequent reduction in the formation of αS plaques in mice [31]. Although it is now well established that βS is able to modulate αS’s aggregation, the mechanisms behind this phenomenon and its exact biological relevance are still under intense scrutiny.

The molecular mechanisms governing the regulatory effect between αS and βS inhibition have also been a source of interesting results, albeit somewhat more divergent: Tsigelny and co-workers were able to create cell-free dimeric and pore-like oligomeric forms of αS and disrupt them with βS [32]; other authors reveal the suppression of initiation and elongation of αS aggregates via competitive binding to surfaces [33]. Similarly in cells, some studies point to the ability of βS to prevent aggregated αS from inhibiting the proteasome, while others suggest that the mechanism involves the reduction in αS expression by increased levels of βS [34]. Recently, two missense mutations of βS have been identified in unrelated cases of DLB–V70M and P123H [35]. These mutations have been shown to promote βS aggregation and accumulation into lysosomal inclusion bodies [36]. Given the regulatory effect that βS exerts on αS aggregation, these mutations could provide valuable information on the interplay between both proteins.

Here, we investigated the effect of βS on different aggregated species of αS, in order to understand which mechanisms define the interplay between these two highly homologous proteins. We developed an experimental method to efficiently coexpress these proteins in a cell-free system, bypassing delicate steps of protein purification and labelling that often irreversibly affect (co)aggregation behaviours, especially in the case of intrinsically disordered proteins. By using single molecule fluorescence techniques, our experimental setup proved robust enough to provide us with valuable insights into the relationship between synucleins.

## 2. Results

### 2.1. Cell-Free Coexpression of αS and βS Allows for Investigation of Their Inter-Regulatory Effect

**Figure 1:**
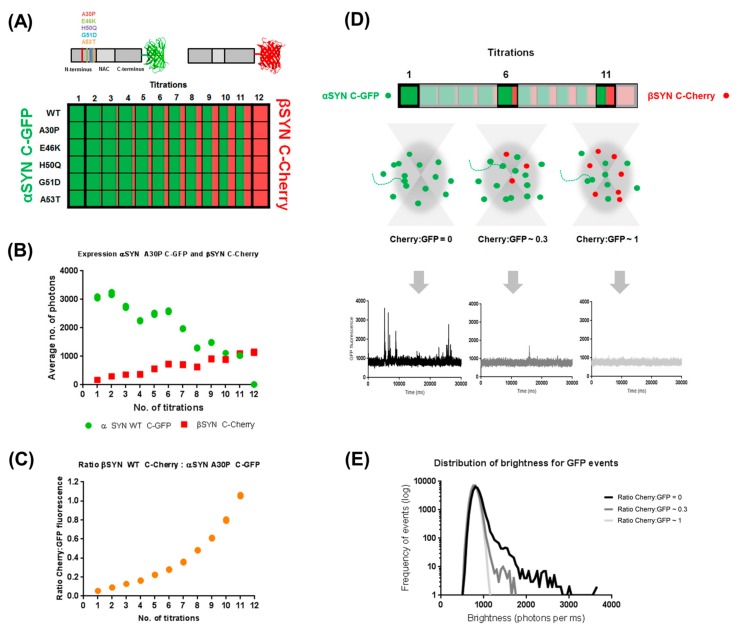
Controlled cell-free co-expression of α- and β-synucleins allows the investigation of their co-oligomerization dynamics. (**A**) Experimental layout for controlled co-expression of C-sGFP-tagged α-Synucleins (WT and five PD-related pathological mutants A30P, E46K, H50Q, G51D and A53T) and C-mCherry-tagged β-synuclein WT. All proteins were expressed in LTE. (**B**,**C**) This experimental layout allows us to accurately control the expression levels of sGFP- and mCherry-tagged proteins and their ratio. (**D**) This translates into different relative amounts of proteins being detected as they diffuse in and out of confocal volume, creating different GFP-fluorescent time traces for each titration between α- and β-synuclein (see examples of titrations 1, 6 and 11). (**E**) Analysis of these traces as a function of brightness (photons per ms) shows the effect of β-synuclein on the oligomerization propensity of α-synucleins.

We first wanted to investigate whether we could design a robust coexpression setup with our cell-free protein expression system to allow for the investigation of the regulatory effect of one protein in the aggregation behaviour of the other. WT and mutant αS and βS were fluorescently tagged in their C-termini using a fast-folding version of GFP (“superfolder GFP” or sGFP) and mCherry, respectively (Figure 1A) and coexpressed in the cell-free translation reaction of the *Leishmania Tarentolae* Extracts (LTE) at different coexpression ratios for 3 h at 27 °C. By titrating the relative amount of DNA template used to prime the LTE, the final levels of expression of GFP- and Cherry-tagged proteins can be varied (Figure 1B). The relative expression levels between the two proteins can be rigorously controlled to obtain a fixed range of expression ratios of βS:αS varying between 0 for the αS C-sGFP controls and ~1 for the last coexpression tested, simulating naturally occurring similar expression levels between these two proteins (Figure 1B,C). To assess the aggregation propensity of αS WT and mutants in the presence of different amounts of βS, we used single molecule spectroscopy. As shown by our group [37], single molecule techniques are well suited to the study of heterogeneous systems.

Briefly, the solution of GFP-tagged proteins at nM concentration is placed on a confocal microscope and fluorescence of GFP is recorded over time. As the fluorescent proteins diffuse in and out of the confocal volume due to Brownian motion, fluctuations around a “monomeric” average intensity are created. Larger fluorescent bursts correspond to larger aggregates, as is the case of the individually expressed αS C-GFP mutants (Figure 1D). The data can then be plotted as a distribution of brightness as in Figure 1E. In these plots, the contribution of the main species is represented as a Gaussian distribution and larger events are creating a tail in the distribution. The length of the tail is correlated with the maximum number of fluorophores in the objects, or to put it briefly, a longer tail indicates the presence of larger aggregates. As βS is titrated into the coexpression system, we observe a reduction of the size of αS aggregates (compare the black and grey curves on Figure 1E) indicating an inhibition of αS aggregation by βS. When a 1:1 ratio of βS:αS is present, αS becomes perfectly monomeric as shown by a typically monomeric fluorescent time trace (Figure 1D), as well as a purely Gaussian distribution of the brightness plot (Figure 1E, light grey curve).

### 2.2. β-Synuclein Is a Selective Inhibitor of Different Aggregation Pathways of α-Synuclein

Having established a suitable experimental setup for assessing the aggregation of fluorescently tagged synucleins, we then set out on systematically investigating how βS affects the behaviour of αS, by using the pathological aggregation-prone αS mutants as a model for rapid access to different aggregated species.

**Figure 2:**
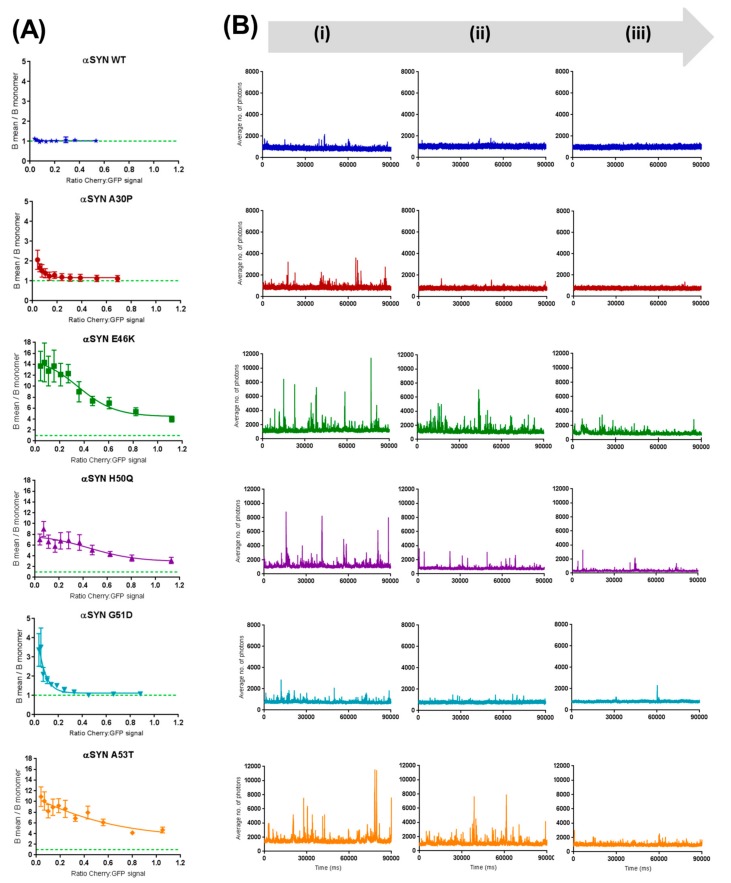
Brightness analysis of α-synuclein mutants reveals selective inhibition of their pathological aggregation by β-synuclein. (**A**) The brightness parameter was calculated for the GFP tagged proteins and plotted against the the mCherry β-synuclein: sGFP α-synuclein ratio (0 < ratio Cherry:GFP < 1.2). For each ratio, three 30 s replicates were acquired and averaged for a total of four co-expression experiments for each protein. The brightness of the GFP monomer is indicated by a green dashed line. Error bars represent the SE of those 4 independent measurements. (**B**) GFP fluorescence time traces show the inhibition of α-synuclein aggregation from (**i**) the initial control C-terminal sGFP-tagged α-syn, to (**ii**) 1:3 of βsyn-mCherry:αsyn-sGFP and, finally (**iii**) 1:1 Cherry:GFP. Arrow indicates this increase in β:α ratio.

As reviewed elsewhere [37] and explained in detail in the “Material and Methods” section, in single molecule spectroscopy, the ‘brightness parameter—B’ is an ideal tool to monitor the aggregation of proteins, representing a measure of the heterogeneity of the sample in a concentration-independent manner. Moreover, when normalized to the brightness of the GFP monomer, B provides information on the size of the aggregated species. By acquiring 30 s fluorescent time traces in triplicate on four different independent measurements for each αS (WT, A30P, E46K, H50Q, G51D and A53T), we were able to generate “aggregation-inhibition curves” across a range of expression ratios C-mCherry-tagged-βS:C-sGFP-tagged-αS between 0 and approximately 1 (Figure 2A). The B parameter therefore complements the fluorescent time traces acquired for these ratios (Figure 2B), all of which paint a clear picture of βS’s chaperoning activity on different forms of αS.

As shown by our group before [22], the controls for each single-point mutant show a segregation into two different groups, with E46K, A53T and H50Q forming larger fibrils, whereas A30P and G51D tend to form smaller objects and WT is mainly monomeric. Remarkably, titration of βS WT into the different coexpression systems results in distinct inhibitory effects. In fact, A30P and G51D “react” much more readily to even low titrations of βS, with very few aggregated forms being detected at βS:αS ratios above ~0.2. Conversely, the fibril-forming mutants E46K, H50Q and A53T are, up to a certain ratio βS:αS, partly unaffected in their aggregation propensity. In fact, even 1:1 βS:αS ratios are not sufficient to rescue these mutants to the monomeric level.

### 2.3. Preferential Binding to Smaller Oligomers Determines a More Efficient Inhibition of Aggregation

**Figure 3:**
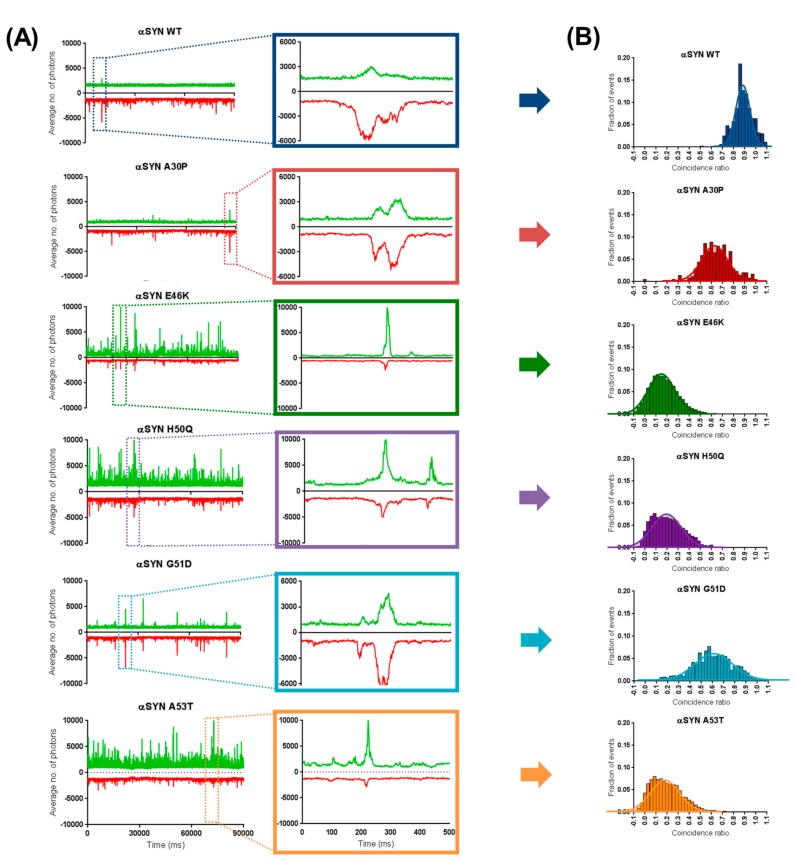
Two-color coincidence unveils the selective mechanism by which β-synuclein inhibits the aggregation of the different α-synuclein mutants. (**A**) For systematic coincidence measurements, only 1:1 (β:α), 90 s traces were selected and three measurements were performed for each protein using a scanning-well microscope approach. Examples of traces and the detailed fluorescent bursts used for coincidence plots show the different trend across the different pathological mutants. (**B**) Coincidence ratios between the traces of mCherry β-syn WT and sGFP α-syns were plotted for a total number of at least 400 events. For each trace, the threshold for an “event” was defined as any burst ≥ average expression + standard deviation of pure GFP monomer.

To explore the mechanism underlying the selective inhibition of some of the αS mutants’ aggregation by βS, we examined the hypothesis that the observed inhibition is the result of different affinities to different aggregated species. To this end, we performed two-color coincidence measurements. In this experiment, two lasers, exciting in the GFP and Cherry wavelengths, are focused in the same confocal volume and fluorescence of the two fluorophores is detected separately. The detection of co-diffusion between GFP and Cherry in the fluorescent bursts indicates the presence of the two species in the same object (Figure 3A). The stoichiometry of the interaction can be quantified as the coincidence ratio ($C=\frac{I_{Cherry}}{I_{GFP}\;+\;I_{Cherry}}$) (Figure 3B).

To understand how this co-diffusion unfolds, all two-color coincidence measurements were first performed at 1:1 ratio of mCherry and sGFP proteins. However, as shown in Figure 2B, in our experimental setup, to work at these βS:αS expression ratios means detecting mostly monomeric form of the C-GFP αS due to the inhibitory effect performed by βS. To solve this problem, the confocal microscope was adapted to automatically scan the wells of the measuring plate during data acquisition and, in this way, retrieve the rarer aggregates that might be missed when relying on Brownian motion. As a result, more aggregates are detected over the course of our 30 s traces, allowing us to collect enough fluorescent events for two-color coincidence measurements.

In respect to the six different forms of αS tested against βS WT, fluorescence time traces show more co-diffusion of mCherry-tagged βS with the sGFP-tagged oligomers of αS A30P and G51D as opposed to with the larger events originated by E46K, H50Q and A53T (Figure 3A). To quantify this effect, all bursts of fluorescence above threshold, corresponding to oligomers, were analysed for coincidence and histograms of distribution of the coincidence ratios (C) were plotted (Figure 3B). These histograms can be used to a measure of the stoichiometry of the interactions. Briefly, a distribution centred on C = 0 indicates the presence of oligomers containing only GFP-tagged proteins while a distribution around C = 1 reflects the presence of Cherry-tagged oligomers/aggregates. The presence of a population at different C values indicates that co-aggregation is possible and the average C value calculates the average stoichiometry of the assembly. The population of A30P and G51D aggregates shows a considerable shift in this average when compared to the other 3 mutants from ~0.6 to ~0.15, respectively. This indicates a clear propensity of βS to co-aggregate with A30P and G51D while being mainly excluded from the E46K/H50Q/A53T aggregates. Indeed, on average, for each A30P/G51D aggregate that diffuses through the confocal volume, a similar number of βS molecules is detected, whereas, for E46K/H50Q/A53T, this number is four times smaller.

**Figure 4:**
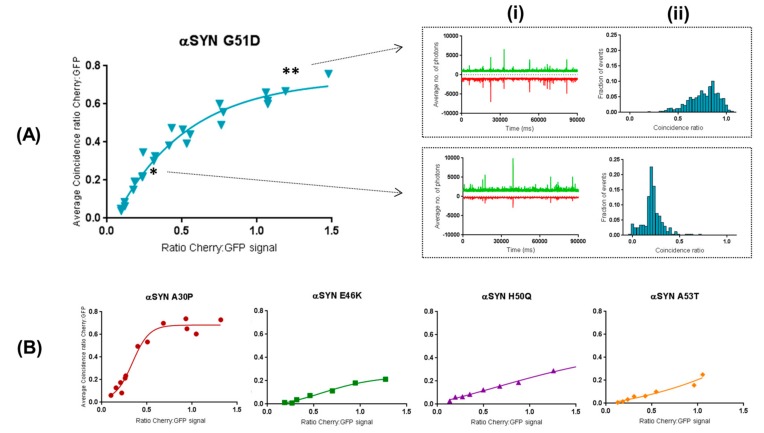
Two-color coincidence applied to the β:α titration range validates affinity differences between aggregated forms of synucleins. (**A**) Two-color coincidence analysis was performed for all 90 s traces (**i**) corresponding to the different concentration ratios tested. The distributions of coincident events (**ii**) were averaged and averages of coincidence ratios were plotted across the β:α ratios as exemplified by αS G51D (“* →” ratio = 0.25; and “** →” ratio = 1.2). (**B**) The same plots were acquired for all mutant forms of αS revealing two types of behaviours.

We then performed the same two-color coincidence analysis to all 90 s traces across the different βS:αS titrations and plotted the averages of the distribution of coincident events against the ratio βS:αS (Figure 4). The threshold for an “event” was defined as the average expression plus the standard deviation of the GFP monomer (Figure S1). Results validated the high propensity for βS to incorporate into aggregates from A30P and G51D, even for low expression levels of βS, whereas other αS aggregated forms are relatively unaffected.

### 2.4. β-Synuclein Does Not Bind to the Aggregates of α-Synuclein Mutants

We next asked whether this co-diffusion reflects co-aggregation with incorporation of βS in αS aggregates during elongation or binding of βS to already-formed αS aggregates.

**Figure 5:**
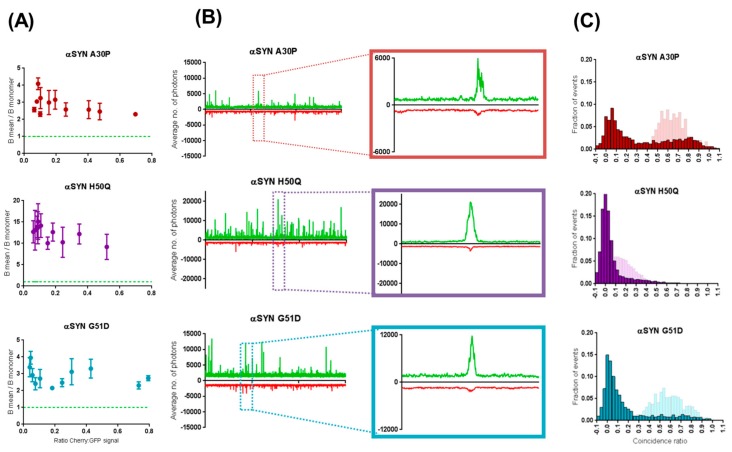
Individual expression of proteins prior to mixing irrevocably reveals a model of selective incorporation of βS into specific αS’s aggregated species. (**A**) Brightness analysis averaged plots were acquired for three independent measurements of triplicated 30 s fluorescent time traces using a stationary microscope plate setup. Error bars represent the SE of those 3 independent measurements Here mutants A30P, H50Q and G51D are shown for final βSWT(Cherry):αS(GFP) ratios < 0.8. Brightness values are normalized against the brightness of the GFP monomer (green dashed line). (**B**) 90-s fluorescent time traces were acquired by scanning confocal microscopy and (**C**) two-color coincidence was applied to a population of >400 fluorescent bursts. Coincidence histograms (dark colour) were compared with the ones obtained from coexpression of the same proteins (lighter).

To test whether this was due to incorporation of βS in αS aggregates, we then individually expressed the same proteins for 3 h before mixing them to obtain similar final expression ratios. Figure 5 depicts the results obtained for three of the five mutants, representing the two groups. Results showed that aggregation propensity (indicated by B) of A30P, H50Q and G51D is relatively unchanged when βS and the mutant α-synucleins are expressed separately (Figure 5A). Furthermore, fluorescent time traces show little coincidence in all cases (Figure 5B), translating into distributions of coincidence ratio shifted towards 0 (i.e., GFP-only objects) (Figure 5C). In particular, we noted a shift in the distribution of coincidence ratios for A30P and G51D as indicated in Figure 5C (top and bottom panels). Established A30P/G51D αS oligomeric assemblies are undisturbed by the addition of βS attesting the inefficacy of βS to modulate αS’s aggregation beyond the earlier steps of oligomerization. These aggregation-prone mutants, here represented by H50Q, do not present this shift in their coincidence plots, indicating that βS plays a limited role in regulating their aggregation cascade. With this approach, we were able to uncover a selectivity of βS to incorporate and modulate further assembly of specific oligomeric forms of αS.

## 3. Discussion

Ever since the discovery of different members in the synuclein family, important research has aimed at understanding how these highly homologous proteins might relate in function. Much evidence has thereafter surfaced supporting the idea that βS plays a role in the inhibition of αS aggregation, but the mechanistic basis and exact stage in αS’s aggregation when this occurs is still unclear. In earlier work, we have demonstrated that single molecule fluorescence methods are a useful tool to quantify protein oligomerization [37] and enable us to dissect the events leading to the formation of fibrils in αS pathology [22]. In the present study, we have investigated the effect of co-expressing WT and mutant β- and α-synucleins in our cell-free system. By applying single molecule brightness analysis and two-color coincidence, we were able to show that βS inhibits the aggregation of αS with different degrees of efficiency depending on the mutant αS tested. In our cell-free system, where we study proteins within a few hours after expression at low concentrations, we observe smaller oligomers formed by A30P and G51D and larger aggregates and protofibrils formed by E46, H50Q and A53T and see segregation into different classes [22]. Therefore, we formulated the hypothesis that the different point mutants represent distinct αS aggregated species and specific oligomerisation/fibrillation steps. Consequently, by investigating how βS modulates the aggregation of αS mutants, we are interrogating its impact on the formation of early oligomers in the aggregation pathway.

**Figure 6:**
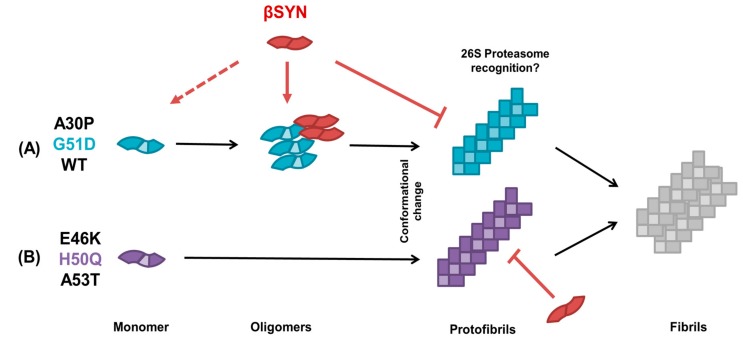
Proposed model of modulation of α-synuclein aggregation by its putative “natural negative regulator” β-synuclein. The model shows two distinct pathways of aggregation for the different αS mutants, in (**A**) and (**B**). αS monomers are depicted as blue and purple coloured blocks with a light-coloured core representing the NAC domain, which is absent in βS, represented by red blocks. Arrows represent the proposed model’s sequence; dashed lines represent discussion points in this model for future studies; capped ends depict no interaction. Monomeric and oligomeric forms are represented unfolded, and a conformational change is required for α-synuclein to display pre-fibrillar and fibrillar species.

Taken together, our data enabled us to construct a proposed model for the chaperoning effect of βS on αS, depicted in Figure 6. Our results point to a more efficient inhibition of aggregation for oligomer-forming A30P and G51D as compared to E46K, H50Q and A53T. This ‘resistance’ to βS’s inhibitory effect shown by the latter class of mutants has not been described before and effectively assigns a selective inhibitory effect of βS towards the earlier steps of oligomerisation in the pathway.

βS and αS seem to be interchangeable in the early oligomers, as shown by the two-color coincidence data of Figure 3 and Figure 4. As the ratio β:α in the system increases, βS replaces αS in the small oligomers in a concentration-dependent manner. The gradual shielding of αS–αS interactions inhibits their self-assembly and ultimately the oligomers of αS can no longer form. At the same time, as βS does not aggregate readily, the overall number of aggregated species decrease with βS concentration.

The inhibition effect occurs very quickly for A30P and G51D, and cannot be attributed to reductions in protein expression levels. In a previous work [22], we have shown that cell-free expressed synucleins mutants displayed aggregation at low concentrations. All mutants showed very stable brightness parameter over an order of magnitude or more, down to 100 nM expression levels. In this work, the co-expression with βS only reduces protein expression by a factor 2 to 3.

One important aspect of our experiments is that αS and βS can co-oligomerize only when co-expressed. As shown in Figure 4, βS can no longer incorporate into the pre-formed αS oligomers. This indicates that co-translational association and fast formation of oligomers is crucial and that oligomers are stable in composition: once trapped in an oligomer, the proteins do not exchange with monomers present in solution.

Our study utilises the relatively large sGFP and mCherry fluorescent tags and we performed extensive checks to make sure that these tags do not interfere with our measurements. This is especially important for synucleins, as the tags are larger than the protein itself. sGFP and mCherry could either block the aggregation of the proteins due to steric effects, or create false-positives interactions if sGFP and mCherry could interact in our experimental conditions. In our previous work [22], we used a much smaller 6× histidine tag and checked that the GFP label did not affect the aggregation propensity of the proteins. We used a Tris-NTA coupled dye to label the oligomers after their expression in our cell-free system and could not find differences in the aggregation properties measured [22]. In the same study, we demonstrated that the sGFP and mCherry tags did not interfere with binding of α-synucleins, as we measured protein–protein interactions using AlphaScreen methods. We also performed coincidence measurements, both at the dimer level and at the oligomer level. This demonstrates that the mCherry tag is not responsible for inhibition of oligomerisation. The complete absence of interaction between WT and some synuclein mutants also proves that the sGFP and mCherry tags do not bind to each other in our experimental conditions, creating false positives. This is expected as these fluorescent tags are now widely used for in cell studies and chaperoning or aggregation effects would have been described.

In our system, we observe that the mutants E46K, H50Q and A53T seem to “skip” the oligomerisation step, or at least transition quickly to pre-fibrillar species. Our data show that βS cannot incorporate efficiently into the aggregates of these mutants, as indicated by the very low coincidence ratios (Figure 3 and Figure 4). We also show that βS cannot efficiently block the aggregation of the E46K, H50Q and A53T mutants as we observe a smaller decrease of aggregation propensity in Figure 2. In that case, αS can fibrillate efficiently on its own, βS is less likely to be recruited, and, logically, βS can no longer interfere with fibrillation and cannot block the elongation of fibrils. In other words, for these αS mutants, aggregation is inhibited as well, but it becomes only significant when the concentration balance between available αS and βS molecules leans to the latter. These findings are consistent with the hypothesis, postulated by other authors, that βS acts a natural retardant of αS aggregation by competing with αS molecules for fibril amplification [33]. To put it simply, our data suggest that βS recognizes the monomeric or oligomeric form of αS but not the fibrillar structure. This is reminiscent of our previous observations that WT αS cannot associate efficiently with the mutants E46K, H50Q and A53T [22]. In many ways, βS behaves as WT αS at the monomeric and oligomeric level.

Other authors have assessed the binding affinity and characteristics of βS WT to αS WT, showing that both directly interact at the monomer level to form transient heterodimers with high specificity [25]. NMR experiments have demonstrated that interactions between the N-terminus of αS and the C-terminus of βS (forming non-propagating heterodimers) are five times stronger and more extensive than those of the C-terminus of αS with its own N-terminus (propagating homodimers that proceed to fibril formation). This could provide us with an explanation as to why the aggregation of A30P and G51D is more efficiently inhibited. Seeing that these two mutants were shown to recruit WT αS [22], if we add βS into the system, competition for binding could determine delay in aggregation. Here, our study of the αS mutants completes the picture of αS–βS interactions.

However, further fundamental biological questions surface from the model we propose here. One would reasonably interrogate whether this putative delay in aggregation exerted by βS also presents an effect in terms of overall toxicity of the ‘equilibrium’ of aggregated species present in the system at any given moment. Moreover, if that is the case, would the regulatory action exerted by βS favour or abrogate toxicity? In fact, oligomers of G51D αS have been shown to be more toxic to cells and the disease caused by G51D mutation to progress faster [38,39]. Recent years have strengthened the views that implicate interactions with proteasome subunits in αS pathology. It has been postulated that βS’s role as a negative regulator of αS is related to a competitive interaction with αS aggregates, which would make them less available for recognition by the 26S subunit of the proteasome system [40]. This could point to a close functional synergy between βS and the degradation pathways in proteostasis, where βS could act as a filter for other degradation pathways. Strikingly, despite the multiple lines of evidence that prove presynaptic colocalization, regulatory effects and interactions between αS and βS, these proteins do not coincide in Lewy body inclusions [41]. Our in vitro data on E46K, H50Q and A53T are consistent with this physiological observation as βS does not associate with their aggregates (Figure 4). If the presence of protofibrils of αS remains to be demonstrated in the brain, our study highlights a differential recognition of αS species by βS and suggests different interactomes for the different aggregation “steps” of αS.

## 4. Materials and Methods

### 4.1. Preparation of LTE

*Leishmania tarentolae* cell-free lysate was produced as described by Johnston and Alexandrov [42,43,44]. Briefly, *Leishmania tarentolae* Parrot strain was obtained as a LEXSY host P10 from Jena Bioscience GmbH, Jena, Germany and cultured in TBGG medium containing 0.2% *v*/*v* Penicillin/Streptomycin (Life Technologies, Carlsbad, CA, USA) and 0.05% *w*/*v* Hemin (MP Biomedicals, Seven Hills, NSW, Australia). Cells were harvested by centrifugation at 2500× *g*, washed twice by resuspension in 45 mM HEPES buffer, pH 7.6, containing 250 mM Sucrose, 100 mM Potassium Acetate and 3 mM Magnesium Acetate and resuspended to 0.25 g cells/g suspension. Cells were placed in a cell disruption vessel (Parr Instruments, Moline, IL, USA) and incubated under 7000 KPa nitrogen for 45 min, and then lysed by rapid release of pressure. The lysate was clarified by sequential centrifugation at 10,000× *g* and 30,000× *g* and anti-splice leader DNA leader oligonucleotide was added to 10 µM. The lysate was then desalted into 45 mM HEPES, pH 7.6, containing, 100 mM Potassium Acetate and 3 mM Magnesium Acetate, supplemented with a coupled translation/transcription feeding solution and snap-frozen until required.

### 4.2. Gateway Cloning System for Cell-Free Protein Expression

α- and β-synucleins DNA were synthesized by IDT (Coralville, IA, USA) as G-blocks and were cloned into the following cell free expression Gateway destination vectors, respectively: C-terminal sGFP tagged (pCellFree_G04) and C-terminal mCherry-cMyc tagged (pCellFree_G08) [45]. Transfer between destination vectors being carried using Gateway PCR cloning protocol, as described in [46].

### 4.3. Cell-Free Coexpression and Fluorescence Spectroscopy of α- and β-Synucleins

Plasmids encoding C-mCherry- and C-sGFP-tagged proteins were expressed in LTE at a concentration DNA template to lysate of 40 nM and 20 nM, respectively, and immediately mixed at different ratios to a final volume of 10 μL across a range of twelve titrations (including two controls, one for individual expression of C-sGFP αS and one for C-mCherry βS). This experimental setup was equally applied to all mutant α-synucleins, aiming at obtaining fixed ratios of expression levels of βS to αS between 0 and 1. Proteins were allowed to coexpress for 3 h at 27 °C and then diluted in buffer A (25 mM HEPES, 50 mM NaCl). A volume of 20 μL of each sample was placed into a custom-made 192-wells silicone plate with a 70 × 80 mm glass coverslip (ProSciTech, Kirwan, QLD, Australia). Plates were analyzed at room temperature on a Zeiss Axio Observer microscope (Zeiss, Oberkochen, Germany) with a custom-built data acquisition setup. In order to gain a clear picture of the oligomerisation and aggregation of the proteins, we acquired time-traces at concentrations between 1 and 100 nanomolar (nM). 

### 4.4. Brightness Analysis

For intensity measurements, the C-terminal sGFP-labelled proteins were expressed. A 488 nm laser beam was focused in the sample volume using a 40×/1.2 NA water immersion objective (Zeiss, Oberkochen, Germany). The fluorescence of sGFP was measured through a 525/20 nm band pass filter, and the number of photons collected in 1 ms time bins (I(t)) was recorded. The proteins were diluted 10 times in buffer A and fluorescent traces were acquired in triplicated measurements of 30 s.

The fluorescent time-trace I(t) obtained shows the presence of intense bursts of fluorescence, with values well over the typical fluctuations of I(t). The presence of these bursts increases the standard deviation of the distribution. To compare the aggregation at different concentrations, we used the Brightness parameter, which is the standard deviation normalized by the average signal, as described previously [37]:

$$B=\frac{SD^{2}}{average}$$

The final brightness parameters acquired for each αS represent averages of four independent measurements. Data was normalized against the brightness determined for sGFP across our working range of concentrations, and curve fitting was performed using GraphPad Prism version 7.00 (La Jolla, CA, USA) for Windows. 

### 4.5. Two-Color Coincidence Measurements

For coincidence experiments, sGFP- and mCherry-labelled proteins were either co-expressed or expressed separately and mixed immediately prior to data acquisition. Fluorescent time traces were acquired for 30 s with a scanning confocal microscope, which scans each well of the microscope plate in different directions for 30 s in triplicate, allowing us to retrieve data on the rarer aggregates that diffuse more inefficiently. For stoichiometric purposes, this procedure was optimized so that final expression levels were 1:1. In this context, average fluorescence was similar in the two channels, and we were able to use the same definition of a “fluorescent event” for both GFP and Cherry. We defined a fluorescent burst as any fluorescence intensity above the sum of the average intensity with the standard deviation of sGFP, acquired for our concentration range. For each event, the intensities of the GFP and Cherry bursts were corrected for background and leakage (6% leakage of the GFP intensity into the Cherry channel).

The coincidence C was then measured as the corrected Cherry signal (I_C_), divided by the total intensity of the burst (C = I_C_/[I_G_ + I_C_]). In the absence of Cherry fluorescence, C is close to zero, while, in the absence of GFP, C tends towards 1. Events with 0.25 < C < 0.75 are considered coincident events. The number of events for each ratio C was counted and normalized to the total number of events to give a probability P(C). Histograms of single-molecule coincidence (P(C) as a function of C) were obtained by measuring >1000 events per interaction, and fitted by Gaussian peaks for GFP-only, coincidence and Cherry-only contributions. The bound fraction was calculated as the proportion of coincidence (0.25 < C < 0.75) to total events. 

## 5. Conclusions

To conclude, these findings attest to the potential for using single molecule techniques to deconstruct an αS aggregation cascade, especially when utilized in conjunction with an experimental design that includes models for different aggregated species along that cascade (pathological mutants of αS) and inhibitors of that process (β-Synuclein).

## Supplementary Materials

Supplementary materials can be found at http://www.mdpi.com/1422-0067/19/2/334/s1.

## Abbreviations

αS	α-Synuclein
αSYN	α-Synuclein
βS	β-Synuclein
βSYN	β-Synuclein
PD	Parkinson’s Disease
CNS	Central Nervous System
DLB	Dementia with Lewy Bodies
IDP	Intrinsically Disordered Protein
BP	Brightness parameter
GFP	Green Fluorescent Protein
sGFP	“superfolder” Green Fluorescent Protein
mCherry	Monomeric Cherry
LTE	*Leishmania Tarentolae* Extract
SD	Standard Deviation
SE	Standard Error
WT	Wild-type
LTE	*Leishmania tarentolae* extract
HEPES	4-(2-hydroxyethyl)-1-piperazineethanesulfonic acid
