# Supplementary material for: Unveiling a Selective Mechanism for the Inhibition of α-Synuclein Aggregation by β-Synuclein
Source: Int J Mol Sci. 2018 Jan 24;19(2):334. doi: 10.3390/ijms19020334 (PMC5855556; doi:10.3390/ijms19020334)
Supplement: Supplementary file 1 [file ijms-19-00334-s001.pdf]

Supplementary Materials

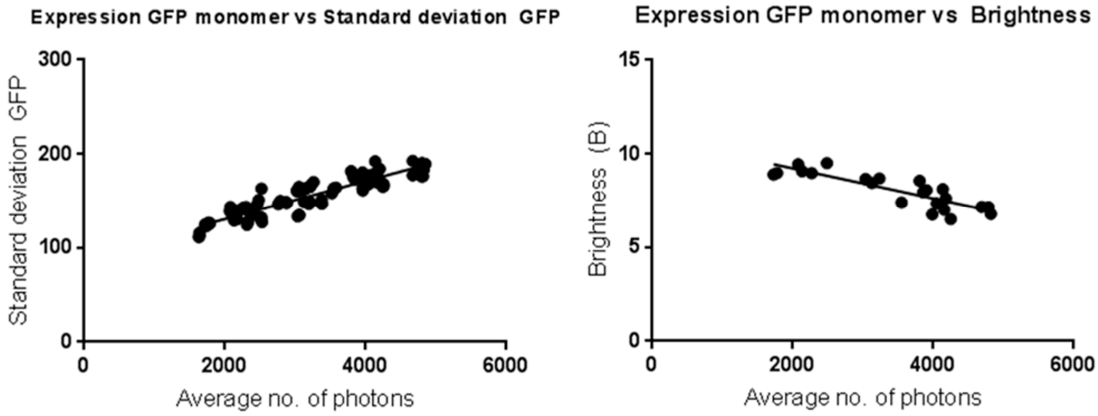

Figure S1. Calibration curves for aggregation studies using GFP-tagged proteins. A monomeric form of the GFP fluorophore was expressed and loaded in increasing amounts on the plate of the confocal microscope. Acquired curves were used to normalize all data: B parameters for GFP-tagged synucleins and for defining a threshold for two-color coincidence experiments (an "event" is any fluorescence detected above the average expression level + standard deviation of the GFP monomer).
